# How to Make Reliable, Washable, and Wearable Textronic Devices

**DOI:** 10.3390/s17040673

**Published:** 2017-03-24

**Authors:** Xuyuan Tao, Vladan Koncar, Tzu-Hao Huang, Chien-Lung Shen, Ya-Chi Ko, Gwo-Tsuen Jou

**Affiliations:** 1 Ecole Nationale Supérieure des Arts et Industries Textiles, 2 allée Louise et Victor Champier, 59056 Roubaix CEDEX 1, France; vladan.koncar@ensait.fr; 2 Taiwan Textile Research Institute, No. 6, Chengtian Rd., Tucheng Dist. New Taipei City 23674, Taiwan; thhuang.1280@ttri.org.tw (T.-H.H.); clShen.0865@ttri.org.tw (C.-L.S.); ycko.1267@ttri.org.tw (Y.-C.K.); gtjou.0219@ttri.org.tw (G.-T.J.)

**Keywords:** wearable textronic device, wash test, ECG device, conductive yarn

## Abstract

In this paper, the washability of wearable textronic (textile-electronic) devices has been studied. Two different approaches aiming at designing, producing, and testing robust washable and reliable smart textile systems are presented. The common point of the two approaches is the use of flexible conductive PCB in order to interface the miniaturized rigid (traditional) electronic devices to conductive threads and tracks within the textile flexible fabric and to connect them to antenna, textile electrodes, sensors, actuators, etc. The first approach consists in the use of TPU films (thermoplastic polyurethane) that are deposited by the press under controlled temperature and pressure parameters in order to protect the conductive thread and electrical contacts. The washability of conductive threads and contact resistances between flexible PCB and conductive threads are tested. The second approach is focused on the protection of the whole system—composed of a rigid electronic device, flexible PCB, and textile substrate—by a barrier made of latex. Three types of prototypes were realized and washed. Their reliabilities are studied.

## 1. Introduction

The “Wearable Technology industry”, as an important sector of the overall “Smart Textile industry”, may be perceived as a newly-defined industrial segment derived from the convergence of many incumbent sectors such as flexible and miniaturized electronics, technical textiles, membranes and barrier insulation, etc. This new industrial sector has numerous fields of applications capturing and emphasizing several key trends such as sports and leisure, healthcare, military and security apparel, fashion, consumer electronics, etc.

According to the 2016 ID Tech Exhibition Report, the Compound Annual Growth Rate (CAGR) will be 37% for smart watches, 34% for medical devices, 146% for smart sports apparel, and 585% for AR&VR (Augmented and Virtual Reality) eyewear devices from 2015 to 2018. In most wearable technology industries, textronic (textile-electronic) structures play an important role as a substrate for sensors and actuators, user-end devices, and communication platforms.

With the development of material science and electronic engineering, wearable textronic devices are tremendously miniaturized and their form moves from bulky and rigid to thin and flexible. More and more devices become invisible and ubiquitous for end-users. The user-machine interfaces become more friendly [1,2,3,4] and the wireless systems are extensively involved in the transmission of vital signs monitoring, motion, etc. [5,6,7,8].

However, the washability issue is always an obstacle in terms of application, reducing the reliability of textronic devices and making them not robust enough, and therefore not ready for the market. Many of experimental wearable textronic devices cannot be used in real life because of the washability problem. Due to the capillary effect, even the hydrophobic textile substrate can still absorb the water in the textile bulk and make electronic devices fail. Besides, the mechanical stresses provoked by the washing process may destroy the electrical contacts between the conductive thread and the electronic wearable device. As a result, the electric impedance becomes uncontrollable after several cycles of the washing process and the wearable device becomes unstable and, in some cases, stops functioning.

In this article, two different approaches aiming at designing, producing, and testing robust washable and reliable textronic systems are presented. The common point of both approaches is the novel method consisting in the use of flexible conductive PCB (printed circuit board), in order to interface the miniaturized rigid (traditional) electronic devices to conductive threads and tracks within the textile flexible fabric and to connect them to antenna, textile electrodes, sensors, etc.

For conductive threads, the polymer based barrier, TPU film (thermoplastic polyurethane), is deposited by the press under controlled temperature and pressure parameters in order to protect the electrical contacts against water and mechanical stresses. The washability of protected conductive thread and the contact resistance between the flexible PCB and conductive thread are studied.

As for the whole textronic device, the latex-based barrier is involved to protect the whole system composed of a rigid electronic device, flexible PCB, and textile substrate. Several prototypes have been designed, realized, and tested to verify the washability and reliability of the protected systems.

Three kinds of prototypes (small LED (Light-Emitting Diode), LED array, and ECG (ElectroCardioGraphy) textronic devices) have been designed, realized, and tested to verify the washability and reliability of the protected systems. Up to 50 standardized washing cycles have been performed to verify the quality and robustness of the barrier.

## 2. Materials and Methods

### 2.1. Conductive Thread and Sewing Technology

Three types of conductive thread have been involved in this study. The silver-plated polyamide thread (Shieldtex 234/34-2 ply HCB, Statex Produktions+Vertriebs GmbH, Bremen, Germany) is sewn into fabrics as transmission wire. Its linear resistance is less than 100 Ω/m, which is acceptable for sensing the ECG signal in low energy consumption system. This two-ply structure allows the thread to be adaptable in the sewing machine or embroidery machine. In order to make electronic components compatible with the textile structure, the flexible PCB, Pyralux^®^ LF9120R (DuPont de Nemours, Contern, Luxembourg), is employed for electrode parts to make the interconnection with the conducting yarn.

**Figure 1:**
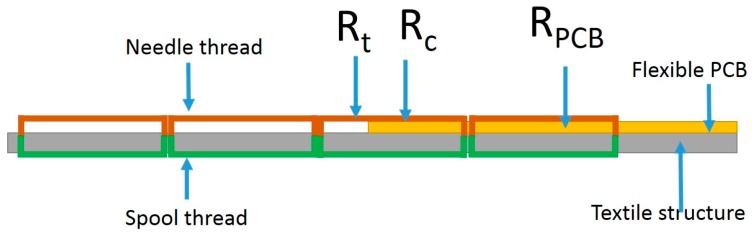
Illustration of the electric resistance model between the conductive thread and PCB on the textile structure.

The interconnection technology between polyamide conductive thread and flexible PCB is realized by using a professional sewing machine (Mitsubishi LS2-190, Mitsubishi, Tokyo, Japan). The conductive thread is used as needle and as spool thread and results in low resistance. The distance between each stitch is 2.5 mm. The electric resistance model of interconnect is shown in Figure 1.

The electric impedance between the conductive thread and PCB is composed of three parts: thread resistance $R_{t}$, contact resistance $R_{c}$, and the PCB resistance $R_{\mathrm{PCB}}$. The PCB electric resistance is as low as negligible compared with the thread electric resistance. According to the contact resistance theory [9], the factors which determine the contact resistance are shown in the equation

(1) $$R_{c}=\frac{\rho }{2}\sqrt{\frac{\pi H}{\mathrm{nP}}}$$

where ρ (Ω·m), H (N/m^2^), n, and P (N) are electrical resistivity, material hardness, number of contact points, and contact pressure between the metal material and the conductive thread interface, respectively. The contact resistance is therefore inversely proportional to the number of contact points and the contact pressure [10].

Two other threads with low resistance (less than 5 Ω/m), the nickel-plated copper wire (Nickel-plated copper wire-12*0.0035 mm nickel plated lines + 100D inelastic yarn, Maeden International Ltd., Taipei, Taiwan) and silver-plated silver copper tinsel (Tinsel AS32JTE20SZ, Maeden International Ltd., Taipei, Taiwan), are used to realize LED application, because the LED application needs a higher current than ECG sensing. These two conductive threads are woven directly into the fabric because of their lack of flexible mechanical properties.

### 2.2. Encapsulation Materials

The thin film of TPU is used to protect the conductive thread and the connection with the flexible PCB. It is welded to textiles by the heat press transfer molding machine (ADKINS BETA, Adkins, Leicestershire, UK). The thickness of TPU was 30 µm. The pressure is 3 bars. The temperature was 180 °C and the pressure time was 20 s. Both sides of conductive thread were protected by TPU film. The electric resistance was measured by the four-point method.

**Figure 2:**
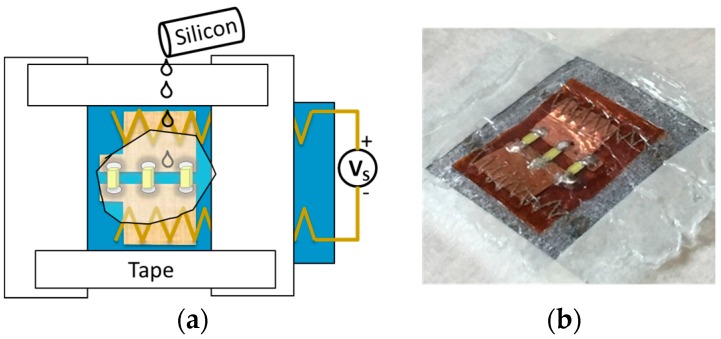
Encapsulation for the LEDs on the textiles. (**a**) The scheme of encapsulation process; (**b**) The photo of encapsulated sample.

Two kinds of silicon were employed to encapsulation the whole textronic devices: Silicon 1 (Super XG-NO.777, CEMEDINE CO., LTD., Tokyo, Japan) and silicon 2 (Rubson Silicone, RUBSON, Boulogne-Billancourt, France). The process of encapsulation is shown in Figure 2. The painter’s tape was employed to make a mask around the LEDs. Then, the silicon was spread on the LED and wiped to make a smooth surface by a chemistry spatula. The samples are dried in an ambient temperature over one night.

### 2.3. Washing Test

The washing test widely used as a reliability test for electronic textiles. For this purpose, ISO 6330 standard was applied to the washability test [11]. It was used by many others for all kinds of components of electronics in textiles [6,12,13]. In this study, ta temperature of 30 °C was used and 50 wash cycles were run. Every wash cycle was programmed for 30 min. The rotation speed was 30 rpm. Between cycles, but not necessarily between every washing cycle, samples were drip dried in a ventilated oven and their functionality was tested. The washing test machine is Datacolor AHIBA IR.

### 2.4. Textile with Three LEDs

**Figure 3:**
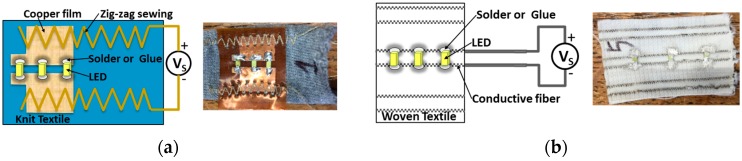
Two types of bases for the textiles with three LEDs: (**a**) The sample with three LEDs by soldering with in on flexible PCB; (**b**) The sample with three LEDs by gluing with silver adhesive on conductive thread.

The LEDs were integrated on textile structures by two different types of connection, soldering with tin and gluing with silver adhesive (Conductive Silver Adhesive #051908-1R, POLYCHEM UV/EB INTERNATIONAL CORP., Taipei, Taiwan) and two types of silicone. Two kinds of conductive thread were employed in the textile structure. The silver-plated polyamide thread was sewn in a zig-zag pattern in order to link the flexible PCB with textile structures (Figure 3a) and the nickel-plated copper wire was woven inside the textile structure without flexible PCB (Figure 3b).

**Figure 4:**
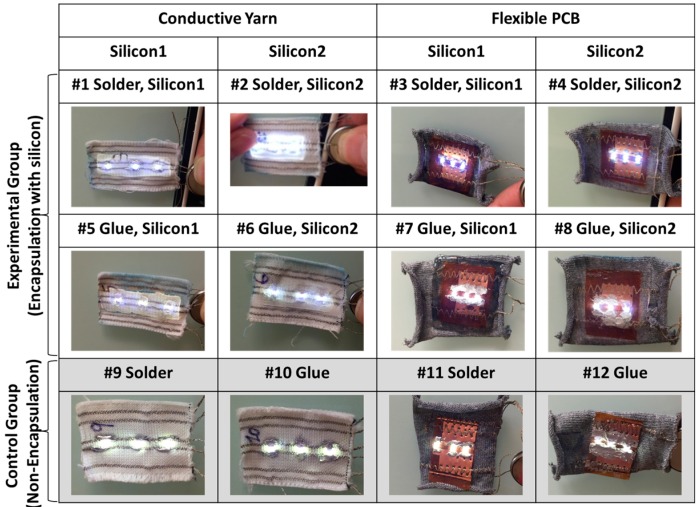
Twelve samples of the textile structures with three LEDs for the washing test. Silicon 1 (Super XG-NO.777) and silicon 2 (Rubson Silicone).

The final samples are shown in Figure 4. Eight different types of samples were realized with two different silicones, two different conductive thread integration methods, and two different LED integration methods. In addition, four types of samples without silicon encapsulation were made in order to conduct a comparative study for the washing tests.

### 2.5. LED Array Device

**Figure 5:**
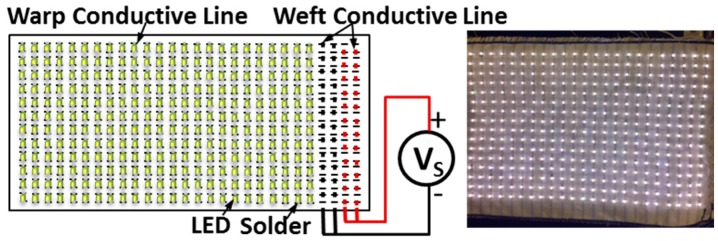
The sample for the textile with LEDs array.

**Figure 6:**
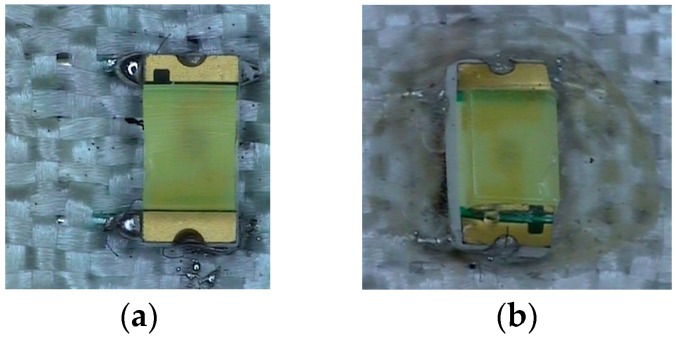
Connection and encapsulation of the textile with LEDs array. (**a**) Solder with tin solder paste and (**b**) encapsulated with Silicon 1 (Super XG-NO.777).

A large-scale sample with 16 × 23 LED array was investigated on a woven structure with silver-plated silver copper tinsel. The LEDs were connected with the warp conductive line and the power lines were connected with the four weft conductive lines. The tin solder paste (LOCTITE GC 10, Henkel Taiwan Ltd., Taipei, Taiwan) was used to glue the LEDs and warp conductive lines and the textile with LEDs array was heated under 230 °C for about 20 s for soldering (Figure 5). After the soldering, the silicon 1 was used to encapsulate the LEDs and the soldered dots (Figure 6).

### 2.6. ECG Monitoring Device

**Figure 7:**
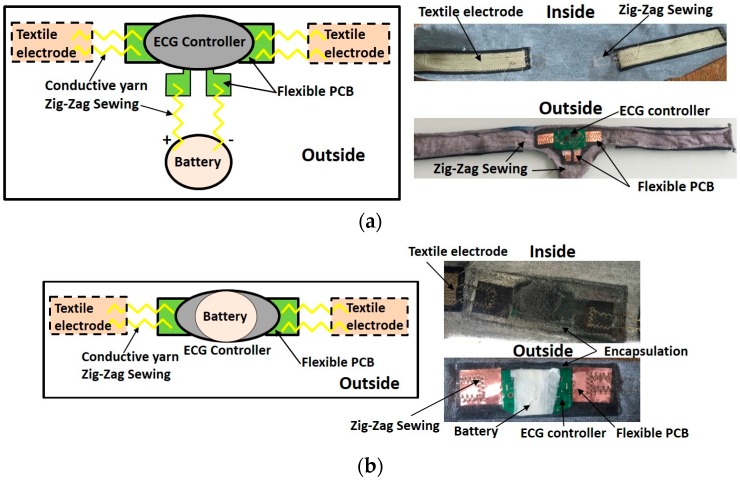
Textiles with ECG device encapsulated with Silicon 1 (Super XG-NO.777): (**a**) Remote battery; (**b**) Embedded battery.

ECG wearable textronic devices are designed to realize smart clothing for monitoring issues. The ECG controller device is made on a rigid substrate. It is connected with the flexible and washable textiles based knitted electrodes, used as skin sensors, realized by TTRI (Taiwan Textile Research Institute) by silver-plated polyamide thread, zig-zag sewing, and flexible PCB. This device is able to monitor heart rate and ECG. It also contains an accelerometer in order to detect the movements. It is equipped with a Bluetooth communication module in order to send data to a smart watch, smart phone, or other communication devices. Two types of samples were designed: battery aside (Figure 7a) and battery embedded (Figure 7b). To achieve the effect of waterproof protection, both sides of the ECG controller and battery were also encapsulated by silicon 1.

## 3. Results and Discussion

### 3.1. Thermoplastic Polymer-Based Barrier for Smart Textile Systems

#### 3.1.1. Washability of Silver-Plated Polyamide Thread

**Figure 8:**
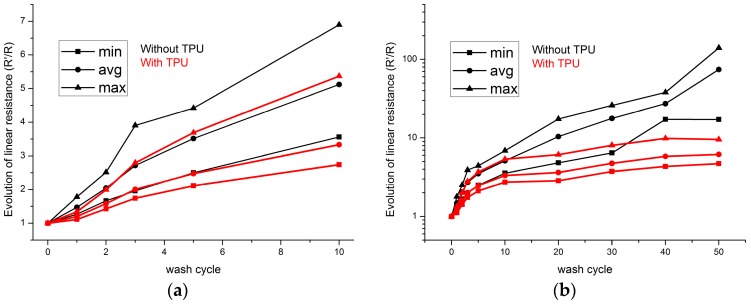
Evolution of electric resistance value of sewn conductive threads: (**a**) From 0 to 50 wash cycles; (**b**) From 0 to 10 wash cycles.

Twenty samples were tested after every cycle of washing tests. Figure 8a shows the evolution of resistance during first 10 cycles of washing tests. All samples possess the linear increase behavior, even for the samples without TPU protection. After 10 wash cycles, the resistances of conductive threads without protection of TPU film exponentially increased with cycles of washing tests. Meanwhile, for the samples with TPU protection, their electric resistances linearly increased with cycles of washing tests. During all washing tests, the resistances of the samples without TPU are always higher than the samples with TPU protection. Figure 8b shows that the resistances for samples with TPU protection arrive at a stable stage and increase lightly; meanwhile the resistances of samples without TPU protection continue to increase.

The absolute values of linear resistance are shown in Table 1. After 50 wash cycles, the average resistance value increases to more than 70 times to the initial value. The deviation of resistance value for samples without TPU is as high as 2773 Ω/m after 50 cycles of washing. As for the samples with TPU protection, the resistance value is in the acceptable range from 51 Ω/m to 307 Ω/m. The deviation is 109 Ω/m after 50 wash cycles. The lower initial resistance value for the sample with TPU can be explained by the increase of contact surface and contact pressure between filaments inside the thread under the pressure of TPU film.

**Figure 9:**
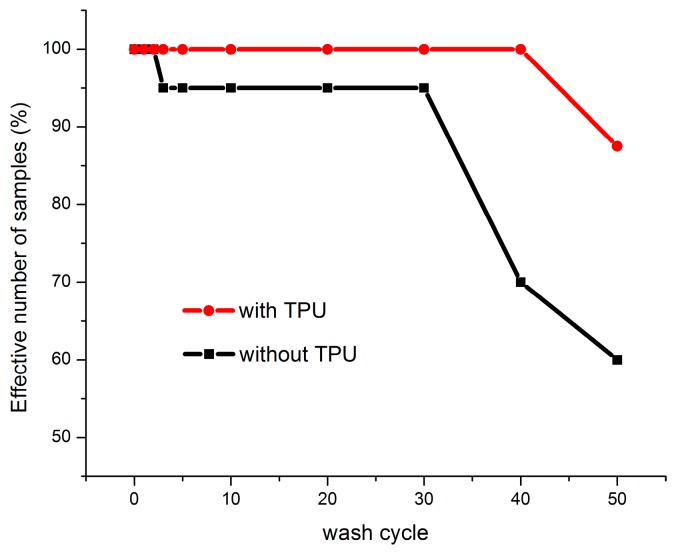
Evolution of effective number of conductive thread samples during washing tests.

The effective numbers of samples are shown in Figure 9. After three washing cycles, resistance values of some samples without TPU cannot be measured. After 40 cycles, 30% of samples were failed. For the samples with TPU protection, all the samples are measurable till to 40 cycles and this result indicates that the samples are better protected by the TPU film.

**Figure 10:**
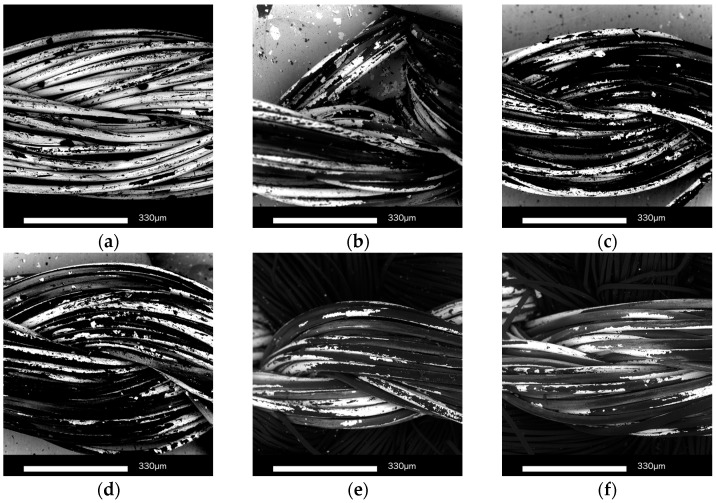
SEM images for sewn yarn without TPU protection during wash tests: (**a**) Before wash test; (**b**) 5 cycles of wash test; (**c**) 10 cycles of wash test; (**d**) 20 cycles of wash test; (**e**) 40 cycles of wash test; (**f**) 50 cycles of wash test.

From the previous studies [14], it is possible to notice that the conductive thread is not affected if the washing temperature is 30 °C. In order to understand the strong increase of the thread resistances, the samples were observed with a SEM. As shown in Figure 10a, peeling-off of plated silver areas can be observed even if the thread is not washed. This failure of coating derives from the mechanical movement during the sewing process. With the increase of the number of washing cycles, more and more plated silvers are destroyed by the mechanical movement (bending, friction, etc.) during the washing (Figure 10b–f). After 50 washing cycles, the silver material can rarely be observed on the outside surface of thread. This may explain the bad conductivity results of washed unprotected threads and the improvements obtained when TPU is used.

It should be mentioned that, even when the high temperature 180 °C was applied during the heat press transfer process, the plated silver on the filament surface was not damaged because of the short operation time.

#### 3.1.2. Washability of Nickel-Plated Copper Wire

**Figure 11:**
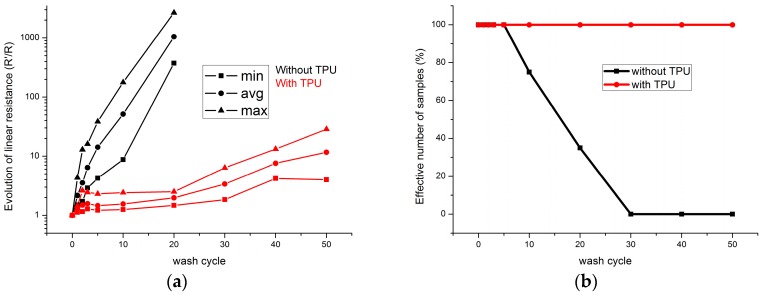
(**a**) Evolution of electric resistance value of sewn nickel-plated copper wire without and with TPU; (**b**) Evolution of the effective number of nickel-plated copper wire samples during washing tests.

Figure 11 shows the evolution of resistance value of the sewn nickel-plated copper wire during washing tests. All samples possess the linear increase behavior at beginning. After five wash cycles, the resistances values of nickel-plated copper wire without TPU exponentially increased with the cycles of washing tests and all of them cannot be measured after 20 washing cycles. Meanwhile, for the samples with TPU protection, their resistance values still increase with the cycles of washing tests and the average resistance is still low. The absolute resistance values are shown in Table 2. After 50 washing cycles, the average resistance value increases to more than 50 times to the initial value. The deviation of resistance value became 173.6 Ω/m for the samples without TPU. As for the samples with TPU protection, the resistance value is in the acceptable range from 3.41 Ω/m to 39.76 Ω/m. The deviation became 23.7 Ω/m after 50 wash cycles.

The effective numbers of samples of the nickel-plated copper wire samples are shown in Figure 9b. After five washing cycles, resistance values of some samples without TPU cannot be measured. After 30 cycles, 100% of samples were failed. For the samples with TPU protection, all the samples are measurable after 50 cycles and this result indicates that the samples are well protected by the TPU film.

**Figure 12:**
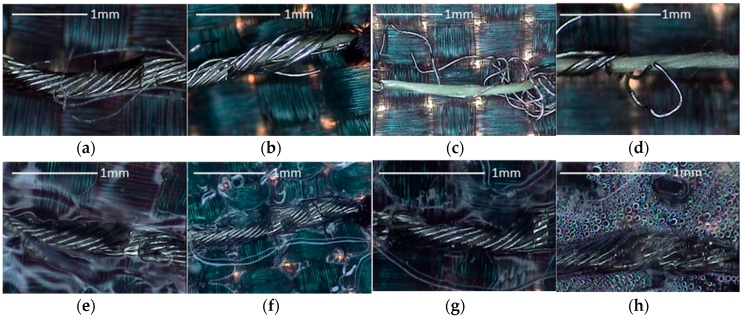
Images for sewn the nickel-plated copper wire without TPU: (**a**) Before wash test; (**b**) 5 cycles of wash test; (**c**) 20 cycles of wash test; (**d**) 30 cycles of wash test with TPU; (**e**) Before wash test; (**f**) 5 cycles of wash test; (**g**) 20 cycles of wash test; (**h**) 50 cycles of wash test.

The images for the nickel-plated copper wire samples during wash testing are shown in Figure 12. For the samples without TPU, the number of broken nickel-plated copper lines increase with the washing cycles (Figure 12b–d). After 50 washing cycles, the silver material can be rarely observed on the outside surface of thread. The break of copper lines results from the mechanical movement during the test. This may explain the bad conductivity results of washed unprotected threads and the improvements obtained when the TPU is used. Meanwhile, for the samples with TPU protection, the nickel-plated copper lines are intact (Figure 12e–h). However, there was some water inside the TPU, showing that the connection of TPU and textile became loose.

#### 3.1.3. Washability of Silver-Plated Silver Copper Tinsel

**Figure 13:**
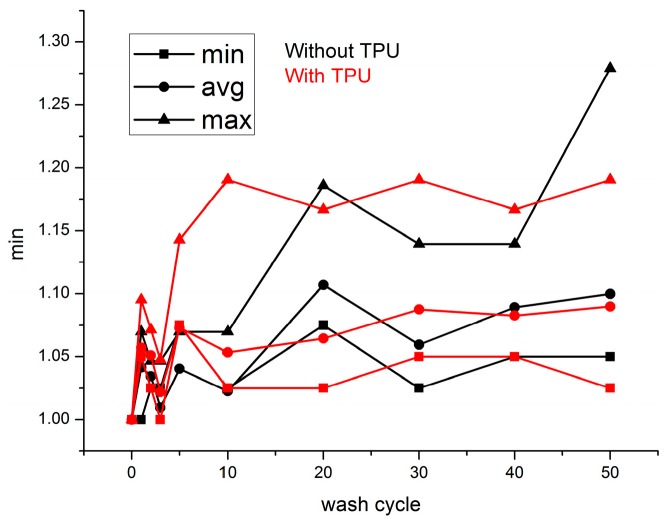
Evolution of electric resistance value of sewn silver-plated silver copper tinsel.

**Figure 14:**
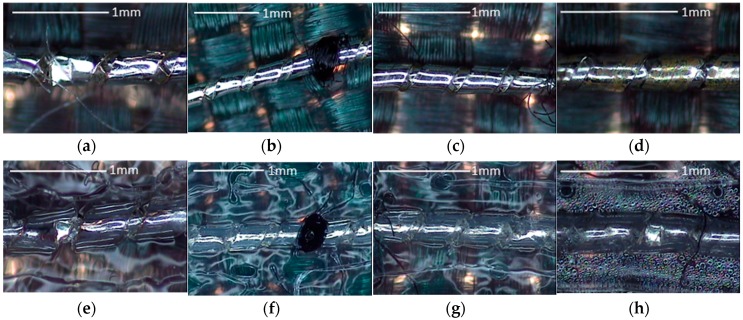
Images for sewn the silver-plated silver copper tinsel without TPU: (**a**) Before wash test; (**b**) 5 cycles of wash test; (**c**) 20 cycles of wash test; (**d**) 50 cycles of wash test with TPU; (**e**) before wash test; (**f**) 5 cycles of wash test; (**g**) 20 cycles of wash test; (**h**) 50 cycles of wash test.

Figure 13 shows the evolution of resistance values of the silver-plated silver copper tinsel during washing tests. The resistance values of all samples are almost constant, even for the samples without TPU protection. The effective number without TPU and with TPU are 100% after 50 washing tests. The average resistance values are from 4.12 Ω/m to 4.49 Ω/m for the samples without TPU and from 4.21 Ω/m to 4.63 Ω/m for the samples with TPU, shown as in Table 3. The result shows the structure of silver-plated silver copper tinsel is strong enough to avoid the destruction provoked by the mechanical stresses during the washing cycles inside a washing machine. Therefore, the resistance values are not significantly different between those of samples without TPU and with TPU. Figure 14 shows images for the washing testing. With or without TPU, the silver-plated silver copper tinsels are all intact in all washing test cycles. However, it is possible to observe that the silver-plated silver copper tinsel is rusty for samples without TPU after 50 washing cycles, as shown in Figure 14d. Therefore, the TPU is still useful for prevention of the rusting.

Comparing to the silver polyamide thread and nickel-plated copper wire, the silver-plated silver copper tinsel has the best resistance performance. Even when there is not any protection, the resistance value is still almost the same as the initial value. However, it is also less flexible than the other two, it is not suitable for the knitted structure, but it can be used in the woven structure for high current applications.

#### 3.1.4. Washability of Interconnections between PCB and Silver-Plated Polyamide Thread

**Figure 15:**
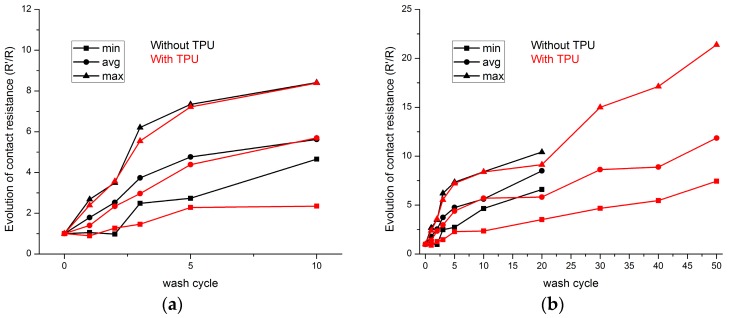
Evolution of electric contact resistance value between conductive thread and PCB: (**a**) First 10 wash cycles; (**b**) All wash cycles.

Figure 15 shows the evolution of contact resistance value throughout washing tests. The contact points and contact pressure between the conductive thread and PCB are reduced because of the mechanical stresses during the washing process. For the first 10 washing cycles, there is little difference between samples with and without TPU protection. Their contact resistance values linearly increase with the number of washing cycles (Figure 15a). The increase of contact resistance value of samples with TPU is lower than the samples without TPU. However, after 10 washing cycles, the samples without TPU are damaged. After 20 washing cycles, all the samples without TPU are destroyed and the contact resistance value cannot be measured (Figure 15b).

The absolute contact resistance values are shown in Table 4. The average values of contact resistance of samples without TPU are almost three times higher than those of samples with TPU. The average value of contact resistance of samples with TPU after 50 washing cycles is less than that of samples without TPU after five washing cycles. This result indicates that the TPU protection improves the contact points and contact pressure between the conductive thread and PCB. As a result, the heat press molding process reduces the contact resistance.

**Figure 16:**
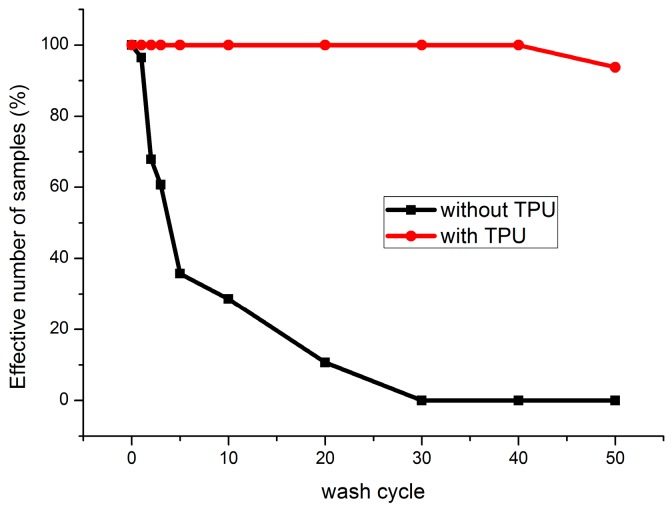
Evolution of effective number of contact resistance samples during washing tests.

Contact resistance with finite values is shown in Figure 16. It is tremendously reduced after two washing cycles. After 30 washing cycles, all the samples without TPU failed. The failure of electric contact for the samples without TPU comes from the fact that the physical contact between the conductive threads and the PCB is loose. This phenomenon can be easily obtained by visual observation. The thread is suspended over the PCB. Meanwhile, the samples with TPU maintain the physical contact between the thread and the PCB. The failure of samples with TPU after 50 wash cycles may be explained by the shedding of plated silver from the surface of thread because the resistance between two PCBs can still be measured even after 50 wash cycles.

### 3.2. Latex-Based Barrier for Wearable Textronic Devices

In order to asses in depth the flexible protective barriers three categories of textile samples have been set up and tested: samples with three LEDs, textiles with LED array and textiles with ECG electronic devices and battery. All the samples have been protected using latex-based barrier (silicon 1 and 2) for smart textile systems in order to make them washable.

#### 3.2.1. Washability of the Textiles with Three LEDs

**Figure 17:**
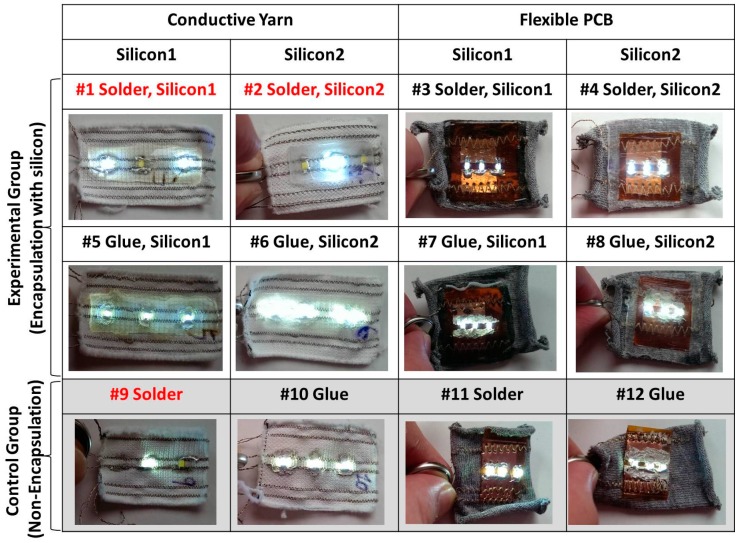
The washing test results after the 18 wash cycles, those that failed are colored red.

Figure 17 exhibits the result after 18 wash cycles. There are failures for samples with LEDs soldered directly on conductive thread from 1 to 18 wash cycles. Sample 1 shows one broken contact LED and Samples 2 and 9 show two broken contact LEDs after 18 washing cycles. This result reveals LEDs soldered to conductive line were easy to break, but the encapsulation still had the effect of reducing the number of broken LEDs.

Compared with Samples 5, 6, and 10, glued LEDs with conductive lines appear to have the better performance than the soldering. This is because the conductive thread is nickel-plated copper wire and the solder joint is brittle for nickel.

Regardless of the woven textile with conductive line or flexible PCB, none of them had a broken LED. This suggests that the flexible PCB combined with the sewing thread is more stable, robust, and could be used to embed the flexible circuit into the textile.

#### 3.2.2. Washability of Textiles with LED Arrays

**Figure 18:**
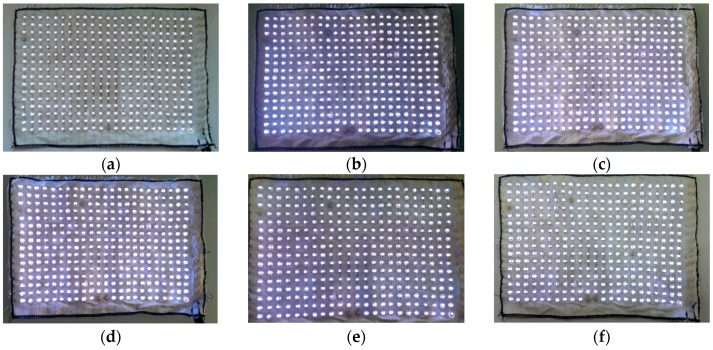
The samples of the textile with LEDs array: (**a**) 5 cycles of wash; (**b**) 10 cycles of wash; (**c**) 15 cycles of wash; (**d**) 20 cycles of wash; (**e**) 25 cycles of wash; and (**f**) 30 cycles of wash.

**Figure 19:**
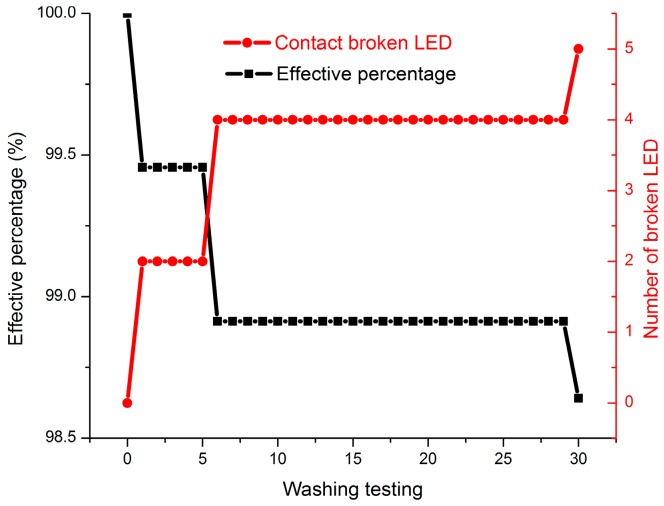
The effective percentage of LEDs from 1 to 30 washing cycles results for the textile LED array.

Figure 18 shows the lighting results after washing test and Figure 19 shows the number of broken LEDs. After 6 wash cycles, 4 broken LEDs were observed; and 5 broken LEDs were observed after 30 wash cycles. It might be because the sample was handmade and some of the LEDs were not soldered well, leading to an early breakage in the washing test. It was thought that the problem might be improved by using an automatic machine for manufacturing. Although there were still 5 broken LEDs found after 30 washing cycles, the encapsulation on the LEDs and LED dots were still in operation and the effective percentage of LEDs can achieve up to 98.6%. In the future, the automatic tin paste extrusion and LEDs placement on the textile should be investigated for mass manufacturing and better production quality.

#### 3.2.3. Washability of Textiles with an ECG Device

**Figure 20:**
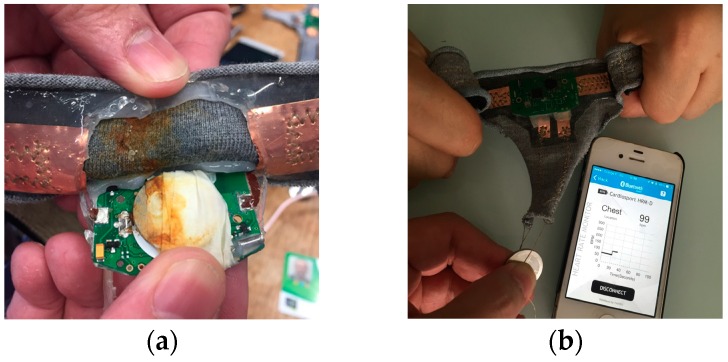
(**a**) The battery-embedded ECG device after five wash cycles; (**b**) The ECG device with a battery after 18 washing cycles.

The result for the textiles with an ECG monitoring device and battery is shown in Figure 20. The battery embedded ECG device still works after four washing cycles. However, after five washing cycles, the device failed because of the oxidized battery (Figure 20a). This shows that the water still affected the battery because of the capillarity effect, even if silicon encapsulation is involved. Whereas, the result for the ECG apart from the battery seems quite satisfactory. The heart rate can be measured after 18 washing cycles (Figure 20b). This implies that the flexible PCB is a promising approach to integrate the electronic device into textile structures. The power supply system is the key point of the washability issue. Future efforts will be focused on this part.

## 4. Conclusions

In this study, the main problem and the most important barrier to market readiness for smart and electronic connected clothing related to its washability has been investigated. The lack of reliability is often due to bad contacts or to an important increase of contact and thread resistance. For the conductive thread, the shedding of conducting material occurs because of the mechanical movement during the washing process. Similarly, an important increase in contact resistance between the conductive thread and the flexible PCB is provoked by the shedding of conductive material on conductive threads.

With thermoplastic polymer film, the conductive threads and their contacts with the PCB can be protected from the mechanical stresses during the washing process. After 50 washing cycles, most of samples were still in operation with an acceptable resistance value. Their conductivity declination became less intensive compared to the samples without film protection.

The flexible PCB with the thermoplastic protection is a promising approach to electronics-in-textile research, which provides a reliable method to integrate electronic components into textile structures.

As for the latex-based barrier in smart textile application, the encapsulation method for LEDs and ECG monitoring devices were found to perform well, which is due to a reliable method to embed the LEDs, flexible PCB, and hardware circuits into woven and knitted structures. The traditional button battery was still not suitable for the washing test. A new kind of textile battery which can endure the washing test should be investigated for a better integration. For e-textile applications in daily life, two approaches (the thermoplastic polymer for the conductive thread and flexible PCB and the latex-based barrier for the rigid PCB or electronic components) can also be combined together for better washability.

## Figures and Tables

**Table 1:** Resistance value during washing tests for the conductive silver thread.

Wash Cycle	Resistance Value (Ω/m)							
	Without TPU				With TPU			
	Min	Avg	Max	Std Dev	Min	Avg	Max	Std Dev
0	62	77	108	13	37	51	96	19
1	81	114	166	24	49	61	114	22
2	112	157	241	31	63	80	140	25
3	129	209	320	54	78	100	167	28
5	158	268	367	49	93	124	218	40
10	228	364	505	92	115	166	293	57
20	309	786	1260	274	120	178	275	51
30	491	1286	1817	471	167	235	405	82
40	1088	1914	2667	484	181	290	522	113
50	1087	5309	10120	2773	208	307	520	109

**Table 2:** Resistance value during washing tests for the nickel-plated copper wire.

Wash Cycle	Resistance Value (Ω/m)							
	Without TPU				With TPU			
	Min	Avg	Max	Std Dev	Min	Avg	Max	Std Dev
0	3.20	3.36	3.60	0.11	3.20	3.41	3.60	0.11
1	4.80	7.29	15.60	2.45	3.60	4.33	5.20	0.51
2	5.50	11.96	46.50	9.79	3.70	5.09	9.40	1.32
3	9.30	21.51	58.00	11.96	4.10	5.37	8.80	1.30
5	13.60	47.81	138.50	41.47	3.90	4.98	8.30	1.01
10	28.00	173.30	640.00	173.60	4.00	5.31	8.70	1.25
20	1200.00	3521.43	9610.00	2794.19	4.70	6.74	9.00	1.37
30	-	-	-	-	5.90	11.52	22.90	5.25
40	-	-	-	-	13.50	25.84	47.60	7.92
50	-	-	-	-	12.80	39.76	103.00	23.73

**Table 3:** Resistance value during washing tests for the silver-plated silver copper tinsel.

Wash Cycle	Resistance Value (Ω/m)							
	Without TPU				With TPU			
	Min	Avg	Max	Std Dev	Min	Avg	Max	Std Dev
0	4.00	4.21	4.30	0.09	4.00	4.12	4.20	0.07
1	4.00	4.38	4.60	0.14	4.20	4.35	4.60	0.11
2	4.10	4.35	4.50	0.10	4.10	4.33	4.50	0.13
3	4.10	4.25	4.50	0.13	4.00	4.21	4.40	0.12
5	4.30	4.38	4.60	0.08	4.30	4.42	4.80	0.13
10	4.10	4.30	4.60	0.14	4.10	4.34	5.00	0.25
20	4.30	4.66	5.10	0.25	4.10	4.38	4.90	0.19
30	4.10	4.46	4.90	0.21	4.20	4.48	5.00	0.20
40	4.20	4.58	4.90	0.22	4.20	4.46	4.90	0.19
50	4.20	4.63	5.50	0.32	4.10	4.49	5.00	0.24

**Table 4:** Resistance value during washing tests for the conductive thread.

Wash Cycle	Contact Resistance Value (Ω)							
	Without TPU				With TPU			
	Min	Avg	Max	Std Dev	Min	Avg	Max	Std Dev
0	0.52	0.90	1.37	0.20	0.19	0.30	0.42	0.07
1	0.92	1.57	2.38	0.34	0.25	0.40	0.58	0.10
2	1.21	2.08	3.03	0.47	0.35	0.68	1.15	0.23
3	1.99	3.17	4.93	0.62	0.29	0.86	1.62	0.36
5	1.99	4.37	5.83	0.94	0.70	1.25	2.18	0.38
10	2.79	5.10	7.17	1.23	0.90	1.63	2.46	0.55
20	7.42	8.76	10.05	0.89	1.05	1.67	3.15	0.55
30	-	-	-	-	1.17	2.52	5.00	1.09
40	-	-	-	-	1.09	2.64	5.71	1.23
50	-	-	-	-	1.63	3.44	6.18	1.33
